# Parasite predators exhibit a rapid numerical response to increased parasite abundance and reduce transmission to hosts

**DOI:** 10.1002/ece3.634

**Published:** 2013-10-10

**Authors:** Skylar R Hopkins, Jennie A Wyderko, Robert R Sheehy, Lisa K Belden, Jeremy M Wojdak

**Affiliations:** 1 Department of Biological Sciences, Virginia TechBlacksburg, Virginia; 2 Department of Biology, Radford UniversityRadford, Virginia

**Keywords:** Commensalism, community dynamics, disease ecology, host–parasite interactions, mutualism

## Abstract

Predators of parasites have recently gained attention as important parts of food webs and ecosystems. In aquatic systems, many taxa consume free-living stages of parasites, and can thus reduce parasite transmission to hosts. However, the importance of the functional and numerical responses of parasite predators to disease dynamics is not well understood. We collected host–parasite–predator cooccurrence data from the field, and then experimentally manipulated predator abundance, parasite abundance, and the presence of alternative prey to determine the consequences for parasite transmission. The parasite predator of interest was a ubiquitous symbiotic oligochaete of mollusks, *Chaetogaster limnaei limnaei*, which inhabits host shells and consumes larval trematode parasites. Predators exhibited a rapid numerical response, where predator populations increased or decreased by as much as 60% in just 5 days, depending on the parasite:predator ratio. Furthermore, snail infection decreased substantially with increasing parasite predator densities, where the highest predator densities reduced infection by up to 89%. Predators of parasites can play an important role in regulating parasite transmission, even when infection risk is high, and especially when predators can rapidly respond numerically to resource pulses. We suggest that these types of interactions might have cascading effects on entire disease systems, and emphasize the importance of considering disease dynamics at the community level.

## Introduction

As global biodiversity continues to decline and anthropogenic disturbances continue to alter ecological communities, there is a growing need to understand how species richness and composition affect the transmission of parasites and pathogens (Keesing et al. 2010). Host biodiversity is often inversely related to parasite transmission across a wide range of disease systems (e.g., the dilution effect; Keesing et al. 2010), although the mechanisms vary according to the details of the disease cycle (e.g., vectors, direct transmission). An increasing number of studies suggest that nonhost species can also alter transmission success of parasites via their interactions with either the hosts or the parasites (e.g., Thieltges et al. 2008a). Several different functional groups of nonhost species have been identified, including competing parasite species, decoy hosts, and predators of either hosts or parasites (Thieltges et al. 2008b; Johnson and Thieltges 2010).

A wide range of nonhost taxa, ranging from aquatic invertebrates to fish to carnivorous plants, are known to consume free-living parasite stages of helminthes, a particularly important group of parasites of humans and wildlife (Johnson et al. 2010; Orlofske et al. 2012). These free-living parasites may be a large component of the zooplankton community in aquatic systems (Morley 2012), and constitute a surprisingly large portion of the total ecosystem biomass (Kuris et al. 2008; Preston et al. 2013). Furthermore, they make an important contribution to energy flow in food webs via direct consumption as prey items by parasite predators and trophic transmission following successful infection in an intermediate host (Lafferty et al. 2006, 2008).

When predators consume free-living parasites as prey, they may impact transmission cycles and alter downstream infection rates of hosts (e.g., Thieltges et al. 2008b). Laboratory experiments have considered the possible reduction in infection provided to downstream hosts by predators of free-living parasite stages by measuring transmission directly (Thieltges et al. 2008a; Orlofske et al. 2012) and by quantifying predator consumption of parasites (Schotthoefer et al. 2007; Kaplan et al. 2009). For instance, damselfly larvae consume free-swimming *Ribeiroia ondatrae* trematode cercariae and thereby reduce second intermediate host trematode infection in tadpoles by as much as 50% under laboratory conditions (Orlofske et al. 2012). These findings contribute to our understanding of the roles that predators of free-living parasite stages play in reducing infection in individual hosts, and thereby how they might impact disease dynamics. However, many basic ecological questions regarding these interactions remain unanswered, including how the densities of predators, parasites, and hosts affect infection. Consumption rates by predators are often nonlinear functions of density, so the answers are likely complex, as illustrated by ongoing debates about the essential role of density in predator–prey interactions (Abrams and Ginzburg 2000; McCoy et al. 2012). Moreover, both short-term consumption rates (i.e., functional response) and the eventual numerical response of the predators will need to be understood in order to truly gauge the importance of predation to parasite transmission.

**Figure 1:**
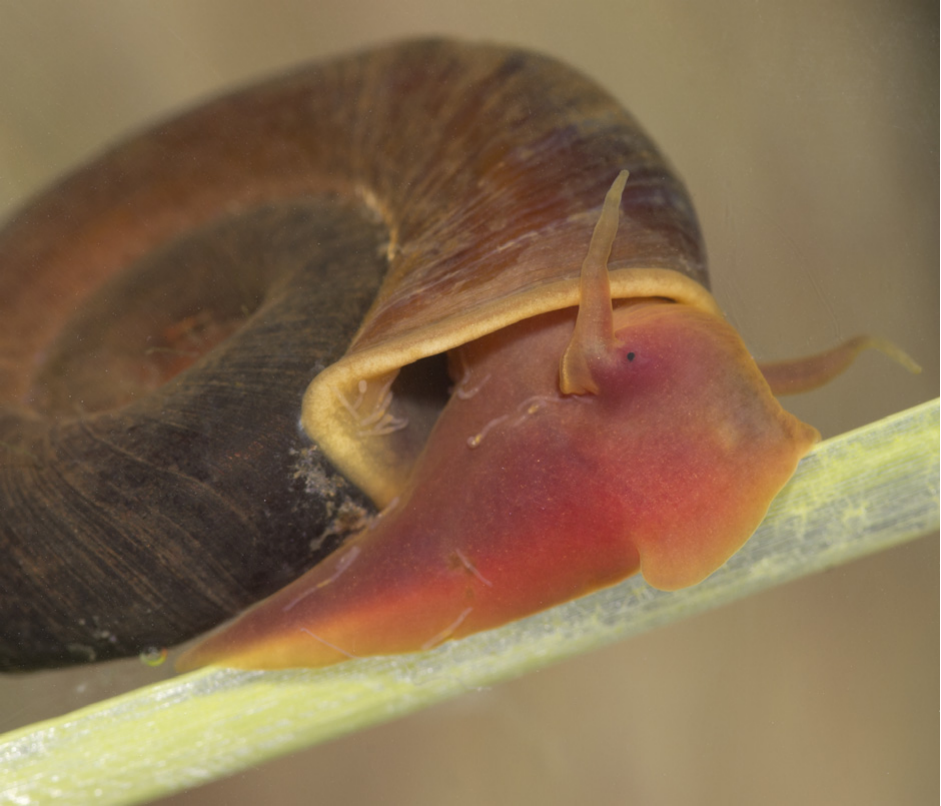
*Chaetogaster limnaei limnaei* worms on the head, foot, and shell of a planorbid snail. Photograph courtesy of Neil Phillips.

To investigate these issues, we focused on the oligochaete *Chaetogaster limnaei limnaei* (hereafter *Chaetogaster*; Fig. 1), a common predator of many ecologically and medically important trematode species (e.g., *Fasciola hepatica*, Rajasekariah 1978; *Schistosoma mansoni*, Michelson 1964). *Chaetogaster* lives as an episymbiont of mollusks, especially pulmonate snails, where it can be found on the head and mantle of upward of 20 host snail genera (Vaghin 1946; Bayer 1955; Gruffydd 1965; Buse 1968; Ibrahim 2007). Laboratory experiments have demonstrated that the consumption of parasites by *Chaetogaster* reduces first and second intermediate host trematode infection in snails (Michelson 1964; Sankurathri and Holmes 1976; Rodgers et al. 2005), and that the number of *Chaetogaster* per snail determines the intensity of trematode infection (Sankurathri and Holmes 1976). Furthermore, there is evidence that over the course of several weeks, *Chaetogaster* populations can increase via asexual budding in response to abundant parasite resources (Shigina 1970; Fernandez et al. 1991). Therefore, this one very common predator of important parasite taxa provides an ideal system for experimentally evaluating the numerical and functional responses of parasite predators.

We performed a field survey to determine first and second intermediate host trematode infection prevalence and intensity among snails with naturally varying *Chaetogaster* infestation intensities. Using the natural range of *Chaetogaster* infestation intensities to parameterize our treatment levels, we then conducted a series of laboratory experiments to evaluate the magnitude of the reduction in infection that *Chaetogaster* provides against second intermediate host trematode infection, under varying density conditions. Specifically, we quantified second intermediate host trematode infection at different *Chaetogaster* and cercarial densities and determined whether the presence of alternative *Chaetogaster* prey (zooplankton) altered infection. Finally, in all experiments, we also quantified the numerical response of *Chaetogaster* to parasite and predator densities.

## Methods

### Study system

Trematodes have complex life cycles, often involving three hosts. For example, the echinostomes in North America, which have been the focus of much recent ecological research (e.g., Fried et al. 2008; Belden et al. 2009; Raffel et al. 2010; Belden and Wojdak 2011), reach maturity and sexually reproduce in the intestinal tract of a mammalian or avian definitive host. Eggs are shed into the aquatic environment in definitive host feces. Miracidia, the first free-living larval stage, hatch within the aquatic environment and actively search for their first intermediate host. We identified the species that we worked with in the experimental portion of this study as *Echinostoma trivolvis* lineage “a” (Detwiler et al. 2010), which uses the snail *Helisoma trivolvis* as a first intermediate host. Within the snail, the miracidia asexually reproduce, ultimately producing thousands of cercariae, the second free-living stage. Cercariae leave the snail host in search of a second intermediate host, such as an aquatic snail (including *H. trivolvis*) or amphibian, in which the cercariae encyst as metacercariae and remain dormant until consumption by the definitive host completes the life cycle. The perpetuation of the life cycle is dependent on many factors, including the transmission success of the free-living larval parasite stages (e.g., miracidia and cercariae). These larval stages must search the environment for their next host while avoiding negative interactions with nonhost species, including predators (Thieltges et al. 2008b).

In the United States and Europe, there are two known subspecies of *C. limnaei*. *Chaetogaster l. vaghini* is considered a parasite, and only occupies the kidney of the snail (Gruffydd 1965). *Chaetogaster l. limnaei* is typically considered a commensal or mutualist of snails (Gruffydd 1965; Rodgers et al. 2005), and was the subspecies used in this study. It remains unclear whether *C. l. limnaei* (hereafter *Chaetogaster*) are detrimental to the host snail. For instance, *Biomphalaria* snail growth and reproduction were not affected by *Chaetogaster* (Rodgers et al. 2005), but *Physa* snail growth and reproduction were reduced when snails were small (3–6 mm) and *Chaetogaster* infestations were high (10 and 20 *Chaetogaster*/snail, Stoll et al. 2013; also see Gamble and Fried 1976). The possibility of negative *Chaetogaster*–snail interactions was not investigated here.

### Field survey

To determine the prevalence and intensity of *Chaetogaster* infestation in the field, and to evaluate potential relationships between *Chaetogaster* infestation and first and second intermediate host echinostome infection, we sampled *H. trivolvis* snails (*n* = 256) from a private pond in Montgomery County, Virginia, on 13 July 2011. The pond has an areal extent of 2.3 ha with an approximate perimeter of 935 m. We haphazardly distributed twenty-three 0.25 m^2^ quadrats within approximately 4000 m^2^ area of the pond, and all *H. trivolvis* within those quadrats were collected, placed individually in 50 mL centrifuge tubes with approximately 30 mL of dechloraminated tap water, and returned to the laboratory. In one area with particularly low snail density, no snails were found by the quadrat method. To obtain some individuals from these low-density areas, all additional snails observed beyond the quadrats were also collected. Snail shell diameters were recorded (nearest 0.01 mm), and then prevalence and intensity of *Chaetogaster* infestation, prevalence and intensity of echinostome metacercariae infection, and prevalence of all first intermediate trematode infections were determined by crushing the snails, counting external *Chaetogaster* under a dissecting scope, and examining wet mounts of the kidney and gonadal tissue at 40× magnification under a compound microscope with darkfield illumination. Prior to processing, snails were maintained at 11°C to prevent snails infected as first intermediate hosts from shedding cercariae and reinfecting themselves with metacercariae.

### Basic experimental procedures

For all experiments, uninfected *H. trivolvis* were collected from stock populations maintained in 1000-L cattle tank mesocosms at Virginia Tech's Kentland Farm, Montgomery County, Virginia, where pre-existing echinostome metacercarial prevalence and intensity were very low (<5% metacercariae prevalence, ∼1 metacercaria per infected snail) and no *Chaetogaster* were present. Snails were individually housed in 120-mL plastic cups containing approximately 90-mL dechloraminated tap water. Snails were fed organic lettuce ad libitum and their water was changed every 2–3 days.

*Chaetogaster* were raised in a single separate 1000-L stock tank containing *H. trivolvis* in which individual snails had up to 200 *Chaetogaster*. The majority of the *Chaetogaster* were located in the mantle cavity (=*C. l. limnaei*; Vaghin 1946; Gruffydd 1965). *Chaetogaster* were collected for experiments by crushing stock tank snails, removing the shells, and rinsing the tissue thoroughly with dechloraminated tap water. The rinse water was then examined under a dissecting scope, and *Chaetogaster* were collected with a pipette and pooled in a single Petri dish. In all trials, *Chaetogaster* were added the day before additional experimental manipulations so that the oligochaetes could infest the snails overnight. Despite rinsing with dechloraminated water, snail hemolymph could not be completely eliminated from the water used to add *Chaetogaster*, so to control for any effects of the hemolymph or chemical signals, one pipette full of water (∼1.2 mL) prepared from crushed snails without *Chaetogaster* was added to each snail in the non-*Chaetogaster* treatments.

*Echinostoma trivolvis* cercariae for experiments were obtained from naturally infected first intermediate host *H. trivolvis* snails collected from a private pond in Montgomery County, Virginia. Cercariae from each snail were morphologically identified as *E. trivolvis* (Fried et al. 1998), and were genetically identified as *E. trivolvis* lineage “a” (see below). Cercariae used in experiments were obtained by placing first intermediate infected snails under incandescent lights for up to 3 h (=maximum age of cercariae used). Cercariae from three to six snails were pooled before distribution across treatments to increase the genetic diversity of parasites used.

After experimental manipulations were complete, snails were left for one additional day after the last cercarial addition to allow metacercariae to develop. Metacercariae and surviving *Chaetogaster* were enumerated according to the above procedures.

### Genetic identification of *E. trivolvis* lineage “a”

Three *E. trivolvis* lineages (a, b, and c), which may represent distinct species with unique host specificity, have recently been identified in North America (Detwiler et al. 2010). For example, *E. trivolvis* lineage “a” appears to use *H. trivolvis* as a first intermediate host, whereas *E. trivolvis* lineages “b” and “c” appear to use *Lynmnaea elodes* as a first intermediate host (Detwiler et al. 2010). In addition, there is some evidence that definitive host use might vary among the three lineages (Detwiler et al. 2012). To determine which lineage we found locally, and to create an easier method for positive identification for future work, we developed a rapid method of molecular identification using restriction fragment length polymorphism (RFLP) analysis of the ND1 gene. Cercariae from individual snails (>15) from this site were collected and stored in 95% ethanol at −20°C prior to DNA extraction. Cercariae were then pelleted by centrifugation for 10 min at 27 k × g and the ethanol was decanted. They were rinsed once in 200 μL low TE (10 mmol/L Tris pH 8.0/0.1 mmol/L EDTA) and centrifuged again as above and the rinse decanted. Samples were then vacuum dried in a Spin-Vac for 20 min at room temperature. DNA was extracted using Gentra Puregene Tissue Kit (Qiagen) scaled to a 200 μL extraction volume. Purified DNA was resuspended in 50 μL low TE. For RFLP analysis, a 530 base pair (bp) fragment of the mitochondrial ND1 gene was PCR amplified using primers developed by Morgan and Blair (1998). PCR amplification was conducted in 50 μL reactions using OneTaq Quick-Load PCR master mix (New England Biolabs, Ipswich, MA) diluted to 1×, 2 mmol/L MgCl_2_, and 0.5 μmol/L each primer with 2 μL of extraction supernatant use to provide template DNA. The thermocycling protocol was 94°C for 3 min, followed by 35 cycles of 94°C for 30 sec, 50°C for 30 sec and 68°C for 1 min with a final extension step of 68°C for 10 min. The amplified ND1 fragment was aliquoted into three 10 μL volumes and each aliquot receive five units of *Alu*I, *Hae*III, or *Msl*I restriction enzyme (New England BioLabs, Ipswich, MA). Restriction digests were incubated at 37°C for 2 h. The resulting fragments were resolved on a 2% agarose gel, and fragment size was estimated using a 100 bp DNA ladder. For all samples, this resulted in 3 fragments of ∼68 bp, 194 bp, and 268 bp when the ND1 PCR fragment was cut with *Alu*I. No observable size difference was seen when comparing the ND1 fragment cut with *Msl*I and *Hae*III and the uncut ND1 PCR fragment. These results are consistent with the samples belonging to *E. trivolvis* lineage “a” based on sequence data reported by Detwiler et al. (2010).

### Experiment 1: *Chaetogaster* density gradient

To determine how *Chaetogaster* infestation intensity affected second intermediate host infection of *H. trivolvis*, individual uninfected snails (*n* = 108, mean shell width [±SD] = 10.90 mm [±1.35]) were randomly assigned to one of five treatments: 0, 3, 8, 15, or 30 *Chaetogaster* added. These *Chaetogaster* densities were based on the natural range found in our field survey, and are well within the ranges reported by other authors for summer months (e.g., Gruffydd 1965; Fernandez et al. 1991). The 0 and 8 *Chaetogaster* treatments were each replicated 27 times, and performed in one trial, while the remaining treatments were replicated 18 times and performed in a separate trial. Ten *E. trivolvis* cercariae were added to each cup every day for 5 days.

### Experiment 2: Cercariae density gradient

To determine whether an abundance of cercariae could overwhelm the reduction in second intermediate host infection provided by *Chaetogaster*, we varied the number of cercariae to which snails were exposed. Individually housed snails (*n* = 68; mean size [±SD] = 9.25 mm [±1.57]) were randomly assigned to one of four treatments: 10, 20, 30, or 40 cercariae per day for 5 days. Five *Chaetogaster* were added to each snail. Nineteen replicates of each treatment were initiated, but we only had enough cercariae to complete 11 replicates of the 40 cercariae treatment for the full 5 days of exposure.

### Experiment 3: *Chaetogaster* and cercariae density

To further evaluate the relationships between *Chaetogaster* infestation intensity, cercarial abundance, and second intermediate host echinostome infection, we performed a 4 × 4 factorial experiment in which we manipulated both the number of *Chaetogaster* per snail (0, 5, 10, or 20) and the number of cercariae to which snails were exposed (0, 10, 30, or 50). Snails (*n* = 145; mean size [±SD] = 9.76 [±1.69]) were randomly assigned to one of the 16 treatments and were exposed to the appropriate number of *Chaetogaster*. The following day, cercariae exposures began and were repeated each day for 3 days total. Ten replicates per treatment combination were initiated, but some replicates were lost from the 50 cercariae treatment when insufficient numbers of cercariae were available, leaving 6, 7, 6, and 6 replicates of the 0, 5, 10, and 20 *Chaetogaster* treatments, respectively.

### Experiment 4: Zooplankton as alternative prey for *Chaetogaster*

Our experimental studies described thus far examined *Chaetogaster* feeding in the absence of alternative prey commonly found in pond ecosystems. To evaluate whether *Chaetogaster* consumption of cercariae is reduced by the presence of an alternative food source, we performed an experiment with zooplankton presence and absence treatments. Uninfected *H. trivolvis* (*n* = 39; mean size [±SD] = 9.66 mm [±1.37]) were randomly assigned to one zooplankton treatment, and each snail was exposed to ten *Chaetogaster*. Zooplankton between 105 and 500 μm were collected by sieving water from a pond on the Virginia Tech campus. *Chaetogaster* are known to consume organisms in this size range (Streit 1977). Five larger zooplankton (approximately three copepods and two daphnids) and ten smaller zooplankton (rotifers) were added to each of the “zooplankton-presence” treatment cups the next day, and within 15 min of zooplankton addition, 30 cercariae were added to each cup. Experimental manipulations were carried out for a single day, rather than three or five, to reduce any impact of algae or nutrients accumulating in the zooplankton presence treatments. We did not quantify cercariae or zooplankton remaining in the cups after experimental manipulation. One replicate of the zooplankton presence treatment was lost due to accident.

### Statistical analyses

All analyses were performed in R v2.15.0 (R Development Core Team 2012). For the field data, negative binomial (NB) distributions were fit to metacercariae and *Chaetogaster* abundance among snails because parasites are often aggregated among hosts (Crofton 1971), with the variance in abundance among individuals greatly exceeding the mean abundance (e.g., overdispersed Poisson distribution; real overdispersion sensu Hilbe 2007). Chi-square goodness-of-fit tests were used to evaluate the fit of these distributions. Fisher's exact tests were used to compare the prevalence of first intermediate host and second intermediate host infection among field snails with and without *Chaetogaster*. A generalized linear model (GLM) with a negative binomial error distribution was used to determine if *Chaetogaster* abundance, infection as a first intermediate host, or their interaction were predictive of metacercariae infection intensity among field-collected snails (function “glm” in package “stats”).

For all laboratory experiments, the assumptions of normal error distributions and homogenous error variances were not reasonable (assessed visually) because again parasites are very often aggregated among hosts. The total number of cercariae in each replicate was known, so we calculated the proportion of successfully encysted cercariae, and used a binomial error distribution. In each case, we added a scaling parameter to account for overdispersion (Crawley 2007). Experiment 3 had two crossed factors and was analyzed as a factorial design. Snail size was initially included in all models, but was never a significant predictor, and thus was dropped from each model. Visual inspection of residual plots and predictions from the models confirmed that our modeling approaches were appropriate. Differences in second intermediate host infection prevalence (the proportion of snails that were infected) were tested with Fisher's Exact tests for Experiments 1, 2, and 4, whereas log-linear (Poisson) regression was used for Experiment 3 because of its factorial design. Linear models with normal error distributions were used to test for differences between treatments in the change in *Chaetogaster* abundance during the experiments, quantified as *c* = ln(*N*_*t*_/*N*_*0*_)/*t*, where *N*_*t*_ is the final abundance, *N*_*0*_ is the initial abundance, and *t* is the time in days. If abundance went to zero (and *c* could not be calculated because log(0) is undefined), the smallest rate of population change that would result in <1 individual by the end of the experiment was used as an estimate of *c* (see Belden and Wojdak 2011 for a similar approach).

## Results

### Field survey

**Figure 2:**
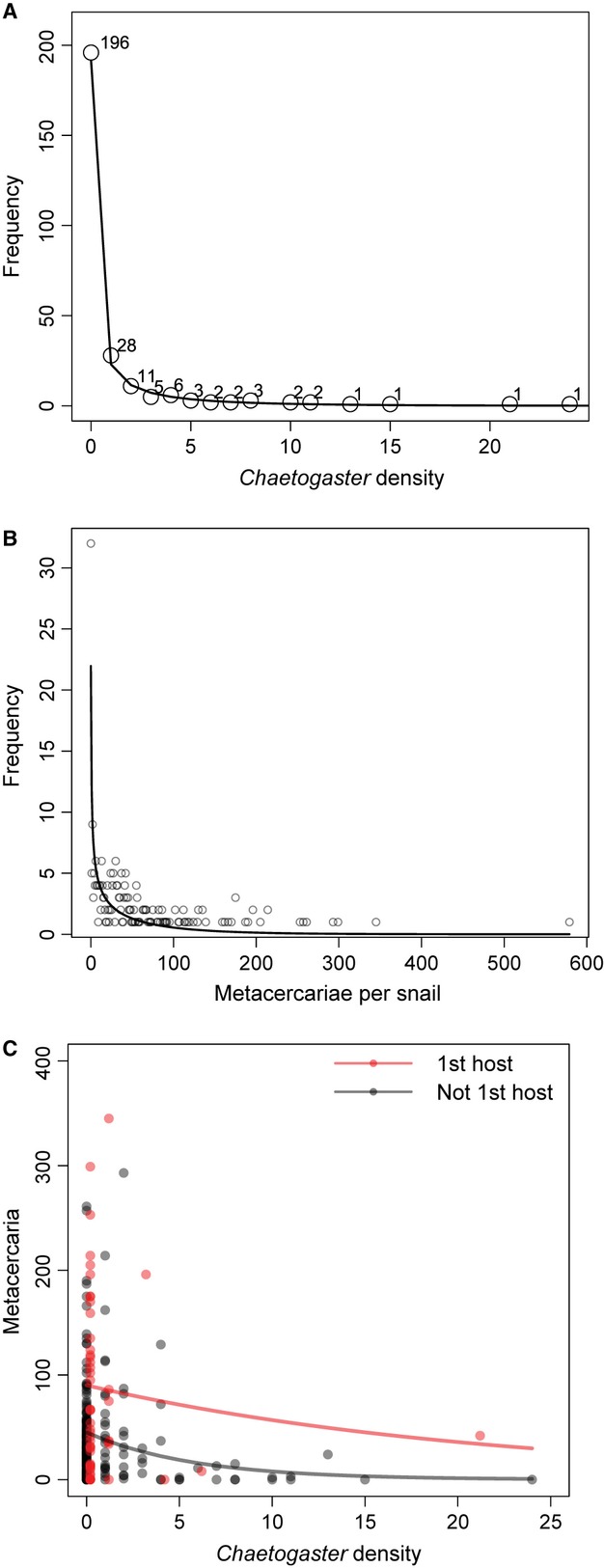
(A) The frequency of observed *Chaetogaster* infestation intensities in *Helisoma trivolvis* snails, (B) the frequency of echinostome metacercariae infection intensities in snails, and (C) the relationship between the number of *Chaetogaster* found infesting a snail, the snail's status as a first intermediate host (infected or not), and the metacercariae infection intensity in those snails, all from a field survey. Lines indicate best-fitting negative binomial models. In (C) red symbols indicate snails infected as first intermediate hosts, and black symbols indicate snails not infected as first intermediate hosts.

We sampled 256 *H. trivolvis* snails from a local pond to characterize their first and second intermediate host trematode infection and *Chaetogaster* infestation. Of these snails, 26% harbored *Chaetogaster*, with an average (±1 SD) of 3.96 (±0.56) worms per infested snail, and 1.02 (*±*0.14) worms per snail overall, including uninfested snails. The distribution of *Chaetogaster* among snails was highly aggregated, with many uninfested snails and some highly infested snails observed, including some snails with >20 worms; the distribution of counts was well approximated by a negative binomial distribution (Fig. 2A; aggregation or clumping parameter from negative binomial, *k* = 0.14; chi-square goodness-of-fit test *P* = 0.63).

Of the field-sampled snails, 21% were infected as first intermediate trematode hosts. Based on morphology and our prior work in this system (Schell 1985; Belden and Wojdak 2011), five trematode taxa/morphotypes were identified, namely an armatae-type, a strigeid-type, *Echinostoma trivolvis*, *Ribeiroia ondatrae*, and *Zygocotyle lunata*, which accounted for 43%, 25%, 14%, 12%, and 2% of the first intermediate host infections, respectively. Two snails were infected with very early, prepatent infections, and the parasites could not be identified. Additionally, 88% of the snails were infected as second intermediate trematode hosts; nearly all the metacercariae were echinostomes, with an average (±1 SE) of 56.6 (±4.58) metacercariae per infected snail, and several snails with more than 250 metacercariae (Fig. 2B). Metacercariae were also highly aggregated among snails; we observed many uninfected and some highly infected snails, similar to that predicted by a negative binomial distribution (Fig. 2B; *k* = 0.54, chi-square goodness-of-fit test *P* = 0.09).

In the field survey, snails with and without *Chaetogaster* were equally likely to be infected as first intermediate hosts (22% and 16% prevalence for *Chaetogaster* present/absent; Fisher's exact test, *P* = 0.356). However, snails with *Chaetogaster* were less likely to be infected as second intermediate hosts than snails without *Chaetogaster* (80% and 91% prevalence for *Chaetogaster* present/absent; Fisher's exact test, *P* = 0.023). Overall, the intensity of infection by metacercariae was negatively related to the abundance of *Chaetogaster* per snail, and metacercariae infection intensity was greater among snails infected as first intermediate hosts (NB GLM; *P* < 0.0001 for both *Chaetogaster* density and first intermediate host infection; Fig. 2C). There was a marginally significant trend toward *Chaetogaster* having less effect on metacercariae infection intensity for first intermediate infected snails (*Chaetogaster* density × first intermediate host infection interaction, *P* = 0.060; shallower slope in Fig. 2C).

### Experiment 1: *Chaetogaster* density gradient

**Figure 3:**
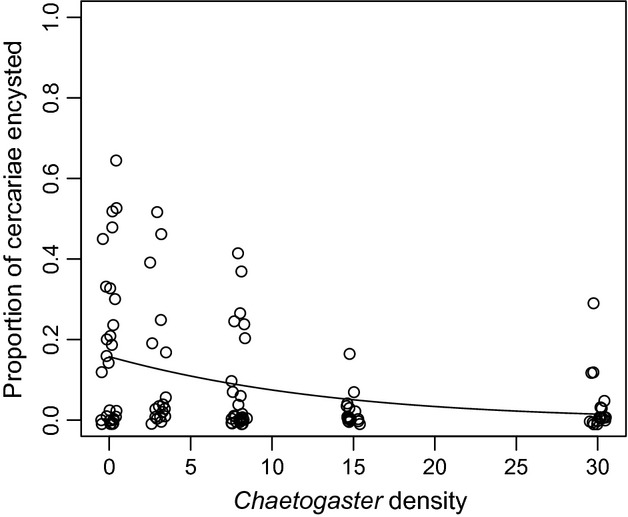
Proportion of cercariae that successfully encysted in snails across a range of *Chaetogaster* densities (Experiment 1). Each point shows an individual snail, and the points were jittered slightly to aid visualization. The line is the predicted fit from the binomial regression model.

Increasing *Chaetogaster* density reduced the proportion of *E. trivolvis* cercariae successfully encysted in *H. trivolvis* snails (Fig. 3, Table 1). Without *Chaetogaster*, 18% of cercariae encysted, whereas with 15 and 30 *Chaetogaster* added, only 2.0 and 3.6% of cercariae successfully encysted, respectively. There was no significant difference in the proportion of snails infected across the gradient of *Chaetogaster* density (Table 2), with 49% infection prevalence overall. However, there was a trend toward reduced prevalence at higher *Chaetogaster* densities; snails exposed to 15 or 30 *Chaetogaster* both had 33% infection prevalence, and snails exposed to 0 or 3 *Chaetogaster* had 63% and 67% prevalence, respectively. *Chaetogaster* abundance decreased through the experiment, with more than 50% mortality on average, and abundance decreased more with increasing initial *Chaetogaster* density treatments (regression slope = −0.00628 [SE = 0.00136], *P* < 0.0001); we observed a −22%, −32%, −57%, and −62% change in population size for the 3, 8, 15, and 30 *Chaetogaster* treatments, respectively.

**Table 1:** Parameter estimates and *P*-values from binomial regression models for Experiments 1–4

Experiment	Parameter	Estimate	SE	*P*
1	Intercept	−1.68	0.21	<0.0001
	*Chaetogaster* density	−0.084	0.026	0.00152
2	Intercept	−2.32	0.43	<0.0001
	Cercariae density	0.0097	0.014	0.496
3	Intercept	−1.44	0.29	<0.0001
	*Chaetogaster* density	−0.061	0.014	<0.0001
	Cercariae density	0.021	0.007	0.00286
4	Intercept	−2.61	0.26	<0.0001
	Zooplankton density	−0.114	0.381	0.766

Parameter estimates are on the logit scale, and can be back-transformed as *e*^x^/(1 + *e*^x^).

**Table 2:** Infection prevalence among snails in experiments, expressed as the percentage of snails infected, with the raw counts of infected/total snails in parentheses

Experiment	Treatment (level)		Prevalence		*P*-value
1	*Chaetogaster* density				
	0		63% (17/27)		0.088
	3		67% (12/18)		
	8		44% (12/27)		
	15		33% (6/18)		
	30		33% (6/18)		
	Overall		49% (53/108)		
2	Cercariae density				
	10		74% (14/19)		0.078
	20		58% (11/19)		
	30		79% (15/19)		
	40		100% (11/11)		
	Overall		75% (51/68)		
3		Cercariae density			
			
	*Chaetogaster* density	10	30	50	0.521
	0	100% (10/10)	80% (8/10)	100% (6/6)	
	5	100% (10/10)	100% (10/10)	100% (7/7)	
	10	70% (7/10)	100% (10/10)	100% (6/6)	
	20	80% (8/10)	80% (8/10)	100% (6/6)	
	Overall		91% (96/105)		
4	Zooplankton density				
	0		65% (13/20)		0.748
	15		58% (11/19)		
	Overall		62% (24/39)		

*P*-values are from Fisher's exact tests (Experiments 1, 2, and 4) and a log-linear regression with Poisson distribution (Experiment 3) comparing infection prevalence among treatments.

### Experiment 2: Cercariae density gradient

**Figure 4:**
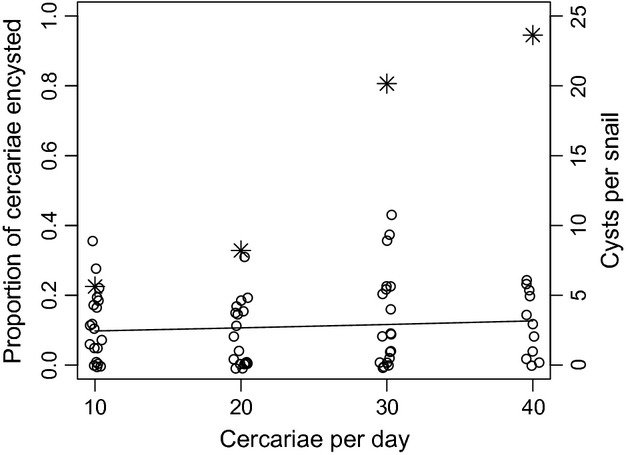
Proportion of cercariae that successfully encysted in snails across a range of *Echinostoma trivolvis* cercariae densities (left *y*-axis; Experiment 2). Each point shows an individual snail, and the points were jittered slightly to aid visualization. The line is the predicted fit from the binomial regression model. The average number of cysts per snail at each cercariae density is indicated with a large star (right *y*-axis).

As cercariae density increased, the proportion of cercariae that successfully encysted remained roughly constant, around 13% (Fig. 4, Table 1). Thus, snails exposed to 40 cercariae had ∼4 times more cysts than those exposed to only 10 cercariae (Fig. 4; second *y*-axis). The proportion of snails that were infected averaged 75% overall, but reached 100% in the 40 cercariae per day treatment (Table 2). *Chaetogaster* abundance increased with increasing cercariae density (regression slope = 0.00500 [SE = 0.00131], *P* = 0.003); we observed a −12%, +9.5%, +29%, and +65% change in *Chaetogaster* abundance for the 10, 20, 30, and 40 cercariae per day treatments, respectively.

### Experiment 3: *Chaetogaster* and cercariae density

**Figure 5:**
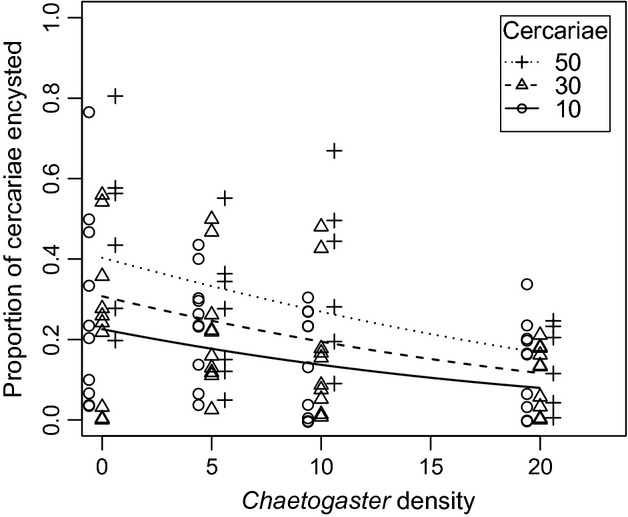
Proportion of cercariae that successfully encysted in snails across a range of *Chaetogaster* densities (Experiment 3). Each point shows an individual snail, and the points were jittered slightly to aid visualization. The three lines are the predicted fits from the binomial regression model for the three cercariae density treatments.

In Experiment 3, we simultaneously manipulated the density of cercariae and *Chaetogaster* using a factorial design. The interaction term (*Chaetogaster* × cercariae) was not significant, so the model was simplified to only contain the two main effects. An analysis of deviance comparing models with and without the interaction (*sensu* Crawley 2007) supported this decision (*F* = 0.54, *P* = 0.463). As in Experiment 1, we observed a significant decrease in proportion of cercariae encysting with increasing *Chaetogaster* abundance (Fig. 5, Table 1). However, as opposed to Experiment 2, we observed a significant increase in the proportion of *E. trivolvis* cercariae successfully encysting in snails with increasing cercariae abundance. Overall, 92% of snails became infected, and there were no differences in infection prevalence between treatments (Table 2). *Chaetogaster* abundance during the experiment again increased with increasing cercariae density (regression slope = 0.00365 [SE = 0.00128], *P* = 0.0057); *Chaetogaster* populations changed by −22%, +6.7%, and +25% in the 10, 30, and 50 cercariae per day treatments, respectively. The change in *Chaetogaster* abundance was not related to initial *Chaetogaster* abundance (regression slope = −0.00405 [SE = −0.00321], *P* = 0.211) nor was there an interaction between initial *Chaetogaster* abundance and cercariae density treatments (analysis of deviance comparing models for final *Chaetogaster* abundance with and without interaction, as above; *F* = 0.0146, *P* = 0.904).

### Experiment 4: Zooplankton as alternative prey for *Chaetogaster*

There was no significant effect of zooplankton density on the proportion of *E. trivolvis* cercariae that successfully encysted in snails (Table 1). Approximately 6.5% of cercariae successfully encysted. There was also no significant difference among zooplankton treatments in the infection prevalence among snails (Table 2), 62% of which were infected overall.

## Discussion

Appreciation for the role of nonhost species in pathogen and parasite transmission is growing rapidly (Thieltges et al. 2008a,b; Johnson and Thieltges 2010; Johnson et al. 2010). Predators often regulate prey populations, and thus predators of free-living parasite stages may control rates of parasite transmission. We observed strong reductions in parasite transmission in a variety of prey and predator density contexts. Moreover, we demonstrated that a particularly ubiquitous predator of ecologically and medically important parasites is able to respond numerically to parasite resource pulses over the course of just a few days. *Chaetogaster* populations increased or decreased more than 60% in response to high prey densities or high conspecific densities, respectively. The literature exploring temporal variation in resources might provide a framework for understanding these predator–prey dynamics (e.g., Yang et al. 2008). Within this framework, the abundance and distribution of parasite predators at any given time should depend on the strength of specialization of the predator on the parasite resource, the ability of the predator to spatially track resource pulses, and the rate of predator population response to a pulse (Ostfeld and Keesing 2000).

**Figure 6:**
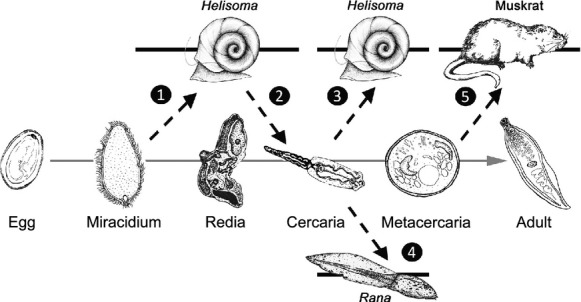
Possible mechanisms by which *Chaetogaster* may alter transmission of *Echinostoma trivolvis*. (1) By consuming miracidia as they attempt to penetrate snails, *Chaetogaster* reduce first intermediate host infection (e.g., Rodgers et al. 2005). (2) By consuming cercariae as they leave snails (e.g., Rajasekariah 1978; Fernandez et al. 1991), *Chaetogaster* might reduce the total number of cercariae entering the aquatic environment. (3) By consuming cercariae as they attempt to penetrate snails, *Chaetogaster* reduce second intermediate infection (this study; Sankurathri and Holmes 1976). (4) By deterring cercariae with their predatory behavior (i.e., Sankurathri and Holmes 1976), *Chaetogaster* might change *E. trivolvis* second intermediate host preference. (5) By changing the intensity of infection in second intermediate hosts (e.g., this study), *Chaetogaster* may change the prevalence and intensity of infection in the definitive host, thereby changing the input of eggs into the system. The gray arrow follows development from egg through adult in the life cycle of *E. trivolvis*. Dashed black arrows indicate movement of larval *E. trivolvis* into or out of a host. Numerals indicate the part of the life cycle impacted by the mechanisms above.

Consideration of numerical responses of parasite predators is necessary to interpret observational studies in the field. If predator numbers can fluctuate rapidly (e.g., weekly) while parasites are accumulated over the lifetime of the host, then field survey “snapshots” (like the one performed in this study) will not capture the complexity of those temporal dynamics and may not reflect the true interdependence of infection and predation processes. For instance, while *Chaetogaster* presence has resulted in consistent reductions in both first and second intermediate host snail infection in laboratory studies (this study; Sankurathri and Holmes 1976; Rodgers et al. 2005; mechanisms 1 and 3, Fig. 6), relationships between *Chaetogaster* abundance and first and second host infection in field studies have been inconsistent (e.g., this study and Sankurathri and Holmes 1976 vs. McKoy et al. 2011 and Zimmermann et al. 2011). We recognize the limited predictive power of the single field survey presented here, and suggest that longitudinal, rather than cross-sectional, field studies will be necessary to track predator responses to pulsed parasite prey.

In addition to considering the role of the numerical response of predators, future work must continue to explore the functional responses of predators to parasite density. The rate of consumption of parasites and thus the rate of host infection should, as we observed here, depend strongly on the relative densities of the predator and prey. As the density of parasites increases, the per capita consumption of parasites by predators should rise and then saturate as each individual spends less time searching for and more time handling its prey (e.g., type-II functional response). The percentage of parasites successfully infecting the host should then increase as parasite abundance increases, exceeding the ability of the predators to intercept parasites. Concordantly, cercariae success increased with cercariae density in our factorial experiment (experiment 3; Fig. 5), which contrasts with the decrease in proportional cercarial success with increasing cercarial density that has often been observed in the absence of parasite predators (Poulin 2010).

However, as predators respond numerically to parasite abundance, the success rate of parasites may drop precipitously as predators become more numerous and each faces a lower per capita density of prey (and thus are less likely to be on the “saturating” part of the functional response curve). The balance between increasing parasite success with increasing parasite density due to saturating functional responses and decreasing parasite success because of numerical increases in predators will depend on the traits and densities of the particular species. In our cercariae gradient experiment (Experiment 2), *Chaetogaster* populations had 5 days to respond to cercariae abundance, and the proportional success of cercariae did not increase with parasite density (Fig. 5). In the factorial experiment (Experiment 3), where cercariae success increased with intraspecific density, *Chaetogaster* had only 3 days to numerically respond. The shorter experimental length may also explain why there was no apparent interaction between initial *Chaetogaster* density and parasite density on snail infection in the factorial experiment. While we cannot say definitively that duration of the experiment caused the differing results, we again emphasize a need to explore the numerical responses of parasite predators further.

One criticism of laboratory experiments of parasite predators has been that nonparasite prey are absent in the experiments, but abundant in the natural environment, and therefore consumption of parasites may be overestimated. However, in the cases where alternative prey were included in experiments, parasite consumption by predators was not reduced (e.g., odonate larvae, and copepods, Schotthoefer et al. 2007; mosquitofish and damselfly larvae, Orlofske et al. 2012). Concordantly, we observed that infection in snails with *Chaetogaster* was no different in the presence of alternative prey, which we might expect if trematodes are a preferred prey type. Free-living trematode stages are likely high-quality prey items because they are energy rich and lack hard-to-digest exoskeletons (Johnson et al. 2010). Individual snails may shed hundreds or thousands of cercariae per day (e.g., Preston et al. 2013), and trematodes may therefore constitute a significant proportion of the zooplankton community (Morley 2012). Correspondingly, a previous study found that in reservoirs with both trematodes and zooplankton, trematodes were 12% of the *Chaetogaster* diet on snails that were not shedding parasites (mechanisms 1 and 3, Fig. 6), and 51% of the diet on snails that were shedding parasites (mechanism 2, Fig. 6; Shigina 1970).

Although the obvious mechanism by which predators of parasites affect disease dynamics is direct consumption of parasites (mechanisms 1–3, Fig. 6), they may play other roles as well. For instance, Thieltges et al. (2008b) described a functional group of nonhost species that reduce infection by causing “physical disturbance” to parasites. In this way, predators of parasites might act to reduce encounter rates between parasites and their hosts by deterring them from certain microhabitats. For instance, Sankurathri and Holmes (1976) found that *Chaetogaster* repelled cercariae from snails and that cercariae would repeatedly circle back toward infested snails until they exhausted their energy stores and died. This might be particularly important in the natural pond environment, especially if deterred cercariae leave the area instead of circling back to the same snail, and end up infecting nearby uninfested snails or other hosts (e.g., tadpoles; mechanism 4, Fig. 6). This mechanism could also contribute to the lower prevalence of second intermediate echinostome infection among snails with *Chaetogaster* that we observed in our field survey, and reinforce aggregation of parasites among relatively few hosts. There is an extensive body of literature devoted to understanding how larval trematodes find susceptible downstream hosts (e.g., Combes et al. 1994; Haas 1994; Haberl et al. 2000), and the distribution of parasite predators in the field adds another layer to consider.

It is not yet possible to predict the net effect of predators on parasites with complex life cycles. For instance, while the immediate impact of consumption of free-living trematode parasites is on first and second intermediate host infection (mechanisms 1 and 3; Fig. 6), these changes might also alter definitive host infection (mechanism 5, Fig. 6), and consequently long-term disease dynamics. Parasite predators may also change the distribution of second intermediate host infection toward species that are more or less likely to reach definitive hosts (mechanism 4, Fig. 6). This would ultimately change the quantity of eggs entering the aquatic environment, and change the exposure of the next generation of first intermediate hosts to miracidia. Given the potentially conflicting magnitudes and directions of these mechanisms (e.g., mechanisms 3 vs. 4, Fig. 6), the net effect of parasite predators on trematode systems may be amplification or dilution of infection risk for definitive hosts, and the direction of the change may depend on the specific trematode system in question (e.g., two vs. three host life cycles, allogenic vs. autogenic life cycles).

The majority of research on how predators of parasites mediate infection dynamics for complex life-cycle parasites has considered a single transmission stage (e.g., Thieltges et al. 2008a; Orlofske et al. 2012), as we have in this study. We stress that these interactions can be density and context dependent, and that parasite predators may cause cascades of indirect effects on infection in downstream hosts. These complexities will be especially important to unravel as scientists consider the use of parasite predators as biocontrols for parasites (e.g., *Chaetogaster* and swimmer's itch, Soldanova et al. 2013). Taking the next steps to consider the net impact of predators of parasites on entire disease systems remains a serious challenge. But without fully understanding the roles that these predators and other nonhost species play in disease systems, we cannot accurately predict how changing biodiversity will impact infectious disease dynamics.
